# Boosting self-efficacy and improving practices for smoking prevention and cessation among South American cancer care providers with a web-based algorithm

**DOI:** 10.1186/s13722-024-00462-w

**Published:** 2024-05-07

**Authors:** Irene Tamí-Maury, Samuel Tundealao, Valeri Noé-Díaz, Esperanza Garcia, Vilma Diaz, Jennie Meier, Mira Dani, Tatiana Vidaurre

**Affiliations:** 1 Department of Epidemiology, School of Public Health, The University of Texas Health Science Center at Houston, 1200 Pressler Street, Suite E641, 77030 Houston, TX USA; 2 Department of Biostatistics, Rutgers School of Public Health, Piscataway, NJ USA; 3 Universidad Intercontinental, Ciudad de México, CDMX México; 4 Instituto Nacional de Cancerología, Bogotá, DC Colombia; 5 Instituto Nacional de Enfermedades Neoplásicas, Lima, Provincia de Lima Perú

**Keywords:** Cancer care providers, Smoking prevention and cessation, Latin america, mHealth, Web-based algorithm

## Abstract

**Background:**

Digital technologies have positively impacted the availability and usability of clinical algorithms through the advancement in mobile health. Therefore, this study aimed to determine if a web-based algorithm designed to support the decision-making process of cancer care providers (CCPs) differentially impacted their self-reported self-efficacy and practices for providing smoking prevention and cessation services in Peru and Colombia.

**Methods:**

A simple decision-making tree algorithm was built in REDCap using information from an extensive review of the currently available smoking prevention and cessation resources. We employed a pre-post study design with a mixed-methods approach among 53 CCPs in Peru and Colombia for pilot-testing the web-based algorithm during a 3-month period. Wilcoxon signed-rank test was used to compare the CCPs’ self-efficacy and practices before and after using the web-based algorithm. The usability of the web-based algorithm was quantitatively measured with the system usability scale (SUS), as well as qualitatively through the analysis of four focus groups conducted among the participating CCPs.

**Results:**

The pre-post assessments indicated that the CCPs significantly improved their self-efficacy and practices toward smoking prevention and cessation services after using the web-based algorithm. The overall average SUS score obtained among study participants was 82.9 (± 9.33) [Peru 81.5; Colombia 84.1]. After completing the qualitative analysis of the focus groups transcripts, four themes emerged: limited resources currently available for smoking prevention and cessation in oncology settings, merits of the web-based algorithm, challenges with the web-based algorithm, and suggestions for improving this web-based decision-making tool.

**Conclusion:**

The web-based algorithm showed high usability and was well-received by the CCPs in Colombia and Peru, promoting a preliminary improvement in their smoking prevention and cessation self-efficacy and practices.

**Supplementary Information:**

The online version contains supplementary material available at 10.1186/s13722-024-00462-w.

## Introduction

In Latin American (LATAM) countries, cigarette smoking is a leading cause of preventable death and disability [1]. In addition to increased morbidity, mortality, and elevated public health risks, smoking use increases economic stress on LATAM countries because of the incredibly high associated medical costs [1]. A 2020 modeling study among 12 LATAM countries estimated that $26.9 billion annual medical costs and approximately 12% of total adult deaths in the region are caused by smoking [2]. About 11.9% of Peruvian adults smoke daily [3], while the smoking prevalence in Colombia is 9.8% [4].

Considerable evidence has indicated that smoking is the most significant risk factor for cancer and continued smoking after cancer diagnosis is associated with poor treatment outcomes and prognosis [5]. Therefore, integrating smoking prevention and cessation services in cancer care is essential for increasing patients’ survival and quality of life [5]. Promptly identifying smoking behavior and delivering smoking prevention and cessation interventions are critical for improving cancer treatment outcomes and survivorship in LATAM [6]. Smoking cessation can improve the overall health and quality of life of cancer patients and reduce the risk of dying from cancer [6]. Additionally, smoking cessation can help cancer treatments work better and reduce the risk of treatment-associated complications [6]. These interventions should be tailored to the needs of cancer patients and survivors, particularly those living in LATAM. However, in many LATAM oncology settings, the lack of awareness and knowledge among cancer care providers (CCPs) about the impact of smoking cessation on cancer treatment outcomes and survivorship, as well as time constraints during clinical visits and limited healthcare resources, make integrating smoking cessation services for cancer patients challenging [7].

Unfortunately, CCPs in low- and middle-income countries (LMICs) such as the LATAM region typically concentrate on cancer diagnosis and treatment due to a lack of personnel trained in smoking prevention and cessation and necessary referral resources [7]. Consequently, they completely or partially dismiss preventive measures like smoking prevention and cessation that could benefit their cancer patients [7]. The training of the CCPs on how to provide smoking prevention and cessation services to their cancer patients is an effective strategy for overcoming these barriers in LATAM [8]. For instance, a study conducted in the United States (US) revealed that CCPs’ integration of tobacco cessation programs into oncology care had a profoundly favorable impact on cancer patients [9]. However, in the LATAM region, there are only a few general smoking prevention and cessation training programs for healthcare providers (HCPs) [10, 11] and only one formal training program for CCPs [8].

The STOP program (Smoking Cessation Training Program for the Oncology Practice), the first and only formal smoking prevention and cessation counseling training program specifically designed for CCPs in LATAM, has proved to increase the trainees’ self-efficacy and practices toward smoking, smoking prevention, and cessation services [8]. However, during the course of the program, STOP trainees reported the recurrent challenge of constantly needing to review the educational materials to provide effective smoking prevention and cessation services to their patients [8]. The use of decision-support algorithms in the healthcare system has increased over time and showed significant potential for determining the best course of treatment for patients [12]. Decision aids and algorithms for cancer screening, prevention, and therapy have been developed for the oncology environment [13]. The use of these guides has been demonstrated to boost decision-making efficiency and knowledge while decreasing decisional conflicts [13]. In the literature, various published scientific research and smoking prevention and cessation algorithms have been used in various clinical settings [14–19].

Mobile health (mHealth)-based technology — a format of medical care delivery and health research based on the use of mobile and wireless devices (e.g., cell phones, tablets, etc.)— has increased in popularity in LMICs because of its widespread accessibility and decreased healthcare costs [20]. This digital transformation has allowed the optimization of clinical care through a more cost-efficient decision-making process where the incorporation of data collected at point-of-care has been key for providing accurate and personalized care [21].

The advancement of mHealth has positively impacted the availability and usability of clinical algorithms through digital technologies [20]. Numerous currently available decisional aids and algorithms have become digitalized due to mHealth [20]. For example, in the smartphone market, over 500 digitalized mobile-based smoking cessation algorithms are available in smartphone applications (apps) where the end user is the patient interested in quitting smoking [22], with only one built exclusively for healthcare providers [23]. There have been reports of some paper-and-pen-based smoking cessation decisional guides and algorithms accessible for HCPs and CCPs in the LATAM region [24–26], but none have been reported to be digitalized.

In an attempt to standardize the overall procedures for providing smoking cessation services in economically deprived oncology settings and increase the self-efficacy of CCPs when assisting their patients during their quitting attempts, our research team proposed the design of a web-based algorithm that was subsequently tested by Colombian and Peruvian CCPs. Therefore, the primary objective of this study was to determine if a web-based algorithm design to support the decision-making process of CCPs differentially impacted their self-reported self-efficacy and practices for providing smoking prevention and cessation services.

## Methods

### Building the algorithm

An extensive literature review of the available smoking prevention and cessation resources was conducted to gather the necessary information to develop the proposed web-based algorithm. These resources included: the Transtheoretical Model (Stages of Change) [27], the US Department of Health Clinical Guidelines for Treating Tobacco Use and Dependence [18], the 5 A’s model [28], published smoking cessation paper-based algorithms used in clinical settings [15], as well as other published scientific literature [14, 16, 17]. Tobacco specialists with clinical and research expertise also contributed to the design of the proposed algorithm. Brief communicatory statements were inserted into the algorithm to help the CCPs initiate and sustain an engaging provider-patient conversation when providing smoking prevention and cessation services to cancer patients.

**Fig. 1:**
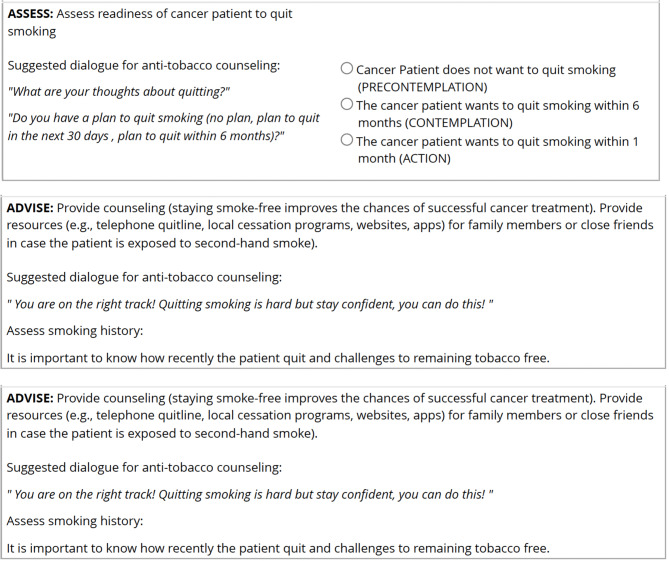
Some selected screenshots from the web-based algorithm

The algorithm was designed with the use of a simple top-down flowchart-like tree structure with multiple possible branches built into the Research Electronic Data Capture (REDCap) platform where the output (personalized smoking prevention and cessation counseling approach and corresponding pharmacotherapy) is based on a set of binary decisions (Fig. 1). Through the utilization of the powerful research electronic data capture of REDCap and its comprehensive data validation features, we were able to ensure the accuracy and integrity of the collected data, enhancing the reliability of the web-based algorithms built upon it. This robust validation process helped reduce errors and biases, ultimately leading to a more precise, robust, and trustworthy clinical decision-making process for smoking prevention and cessation.

The web-based algorithm was initially developed in English and subsequently adapted culturally and linguistically to Spanish. We used neutral Spanish to minimize regional variations and dialectical differences among CCPs in Colombia and Peru. The ultimate goal of this adaptation was to foster engagement and empowerment among CPPs making informed choices aligned with their cultural values. The algorithm was pre-tested among 10 CCPs (5 in each country). Feedbacks from these ten individuals were key for refining the design of the web-based algorithm before testing its usability and acceptability in a larger study sample.

### Participants, setting, and procedure

Twenty-nine CCPs from the National Cancer Institute in Colombia (INC-Colombia) and 24 from the National Institute of Neoplastic Diseases in Peru (INEN) were invited to pilot-test the web-based algorithm three months after completing a smoking prevention and cessation training program that covered the epidemiology of tobacco use in LATAM, cancer and other diseases associated with cigarette smoking, behavioral-based smoking prevention and cessation interventions, and cessation medications and therapies. The sample size for this pilot study was dependent on the sixty CCPs (30 at INC-Colombia and 30 at INEN) who were originally invited to participate in the STOP training program for smoking prevention and cessation. This convenience sample was considered appropriate by the leadership at INC-Colombia and INEN to avoid any disruption in their daily workflow and clinical activities.

The inclusion criteria for this study were (1) being appointed at one of the above centers; (2) ≥ 18 years old; (3) had direct contact with cancer patients; and (4) proficiency/access to internet with a computer, tablet, or smartphone.

Before implementing the study, an introductory 1-hour virtual meeting was conducted to educate CCPs about the functionality and use of the web-based algorithm. Afterward, participants completed an online pre-test data collection form. The link and quick response (QR) code for accessing the algorithm were distributed via email. Additionally, printed cards with the QR code were provided to CCPs to facilitate their access to the algorithm.

All study participants were asked to use the web-based algorithm with at least ten patients every month for a total period of 3 months, after which the study participants completed an online post-test form. The pre-test and post-test were identical, collecting information about demographics (e.g., age, sex assigned at birth, level of education, profession, and time devoted to patient care), as well as CCPs’ self-efficacy, and practice for smoking prevention and cessation:

- ***Self-efficacy domain***: The questions (SI appendix, Table S1) included in this domain were adapted from the International Association for the Study of Lung Cancer (IASLC) survey [29] and the National Health Interview Survey (NHIS) [30]. This domain had seven questions and assessed the CCPs’ self-efficacy to provide smoking prevention and cessation to their cancer patients.
- ***Practice domain***: The practice domain was also adapted from the IASLC and NHIS surveys and had six questions [29, 30]. This domain assessed the smoking prevention and cessation practices of the CCPs (SI appendix, Table S2).
- ***Usability***: We used the System Usability Scale (SUS) to measure CCPs’ usability perception of the web-based algorithm [31]. The SUS is a widely used metric comprising 10 standard questions on a 5-point Likert scale from *Strongly Agree* to *Strongly Disagree* (Table 1). The web-based tool or system with a benchmark cut-off score equal to or greater than 68 is perceived to have a high usability.

**Table 1:** The system usability scale

	System usability scale
1	I think that I would like to use this web-based tool frequently.
2	I found the tool unnecessarily complex.
3	I thought the tool was easy to use.
4	I think that I would need the support of a technical person to be able to use this tool.
5	I found the various functions in this tool were well integrated.
6	I thought there was too much inconsistency in this tool.
7	I would imagine that most people would learn to use this tool very quickly.
8	I found the tool very cumbersome to use.
9	I felt very confident using the tool.
10	I needed to learn a lot of things before I could get going with this tool.

After completion of the post-test at three months, CCPs were invited to participate in a focus group to share their perceptions about the usability and acceptability of the web-based algorithm. A total of 4 focus groups were conducted (2 in Colombia and 2 in Peru) at the INC-Colombia and INEN facilities by a trained moderator who asked open-ended questions about CCPs’ views of the algorithm and requested feedback on specific design elements of the web-based tool. Peruvian data were collected in April 2019, while Colombian data was collected in September 2019. Each focus group lasted less than 60 min. Participants were informed of the study procedures, risks, and benefits and provided written consent to participate in the focus group discussions and for the audio recording. All focus groups were digitally recorded, transcribed verbatim, de-identified, translated to English, and saved as Microsoft Word files.

### Analysis plan

#### Demographic information

Descriptive statistics were used to summarize the CCPs’ demographic characteristics. Bivariate analyses between the Colombian and Peruvian CCPs’ demographic characteristics were computed using the Chi-squared test (or Fisher’s exact test, when appropriate) for categorical variables and a two-sample t-test for the continuous variable.

### Quantitative data

- **Self-efficacy**: Self-efficacy questions also had responses on a 5-point Likert scale and a score range of 7–35. Change in the CCP’s smoking prevention and cessation efforts self-efficacy was evaluated using the Wilcoxon signed Ranked test. The effect size of the change in self-efficacy was computed as the absolute value of the difference between the post-test and pre-test self-efficacy scores divided by the mean of the standard deviations for each.
- **Practice**: The practice domain had questions with responses on a 5-point Likert scale and a possible score range of 6–30. We used the Wilcoxon signed Rank test to measure a change in the CCP’s smoking prevention and cessation practice before and after using the web-based algorithm. The effect size of the change in practice was calculated as the absolute value of the difference between the post-test and pre-test practice scores divided by the mean of the standard deviations for each.
- **Usability**: Each CCP’s SUS score was calculated and then converted to percentile ranks. The SUS scores of the CCPs were combined, and the mean and standard deviation were calculated for each and both countries.

The significance threshold was set at 0.05. All quantitative analyses were conducted using Stata v. 17.0/SE (StataCorp, College Station, Texas).

### Qualitative data

Two coders performed a round of coding on the four transcripts. Initially, each coder reviewed and coded the transcripts individually. Through this, a preliminary list of codes was established. Finally, both coders collaborated on subsequent pass coding processes where all transcripts were reviewed thoroughly, and a list of codes was refined based on the inter-coder agreement. Both coders established agreement through subjective discussion throughout the final coding process. Instances of disagreement were resolved through coder discussion, a revisiting of the transcripts and notes, and recoding to encompass both coders’ perspectives if appropriate. A thematic analysis was performed manually to identify the major themes that reflected the acceptability and usability (i.e., reported merits, flaws, and suggested modifications) of the web-based algorithm tested by the CCPs.

### Ethical statement

The study was approved by the INC in Colombia (Cod INC-STOP Protocol/EX- 00812), the University of Texas MD Anderson Cancer Center Houston (Protocol No. PA17-0878), and the University of Texas Health Science Center Houston (Protocol No. HSC-SPH-20-1339). Protocol approval was not required from the INEN in Peru as the STOP program was an educational effort. Signed informed consent was obtained from all participating CCPs.

## Results

### Demographic characteristics of the CCPs

The average age of the CCPs was 35.6 (± 8.38) years. There were six male CCPs and 47 female CCPs. About 60% of the CCPs had college degrees, while the remaining were either specialists or held a master’s or Ph.D. More than 80% of the CCPs devote more than 50% of their time to patient care. The sociodemographic characteristics of the CCPs at each participating cancer center are described in Table 2.

**Table 2:** Sociodemographic characteristics of the CCPs

	Total (n = 53)	Colombian CCPs (n = 29)	Peruvian CCPs (n = 24)	*p*-value
**Age** (Years)	35.6 (± 8.38)	35.0 (± 8.93)	36.4 (± 7.78)	0.557^a^
**Gender**				*0.027* ^*b*^
Male	6 (11.3%)	6 (20.7%)	0 (0.0%)	
Female	47 (88.7%)	23 (79.3%)	24 (100.0%)	
**Highest Level of Education**				0.097^c^
College	31 (58.5%)	14 (48.3%)	17 (70.8%)	
Post College, such as specialization, master’s, PhD	22 (41.5%)	15 (51.7%)	7 (29.2%)	
**Profession**				0.912^b^
Nurses	52 (98.1%)	28 (96.6%)	24 (100.0%)	
Physicians	1 (1.9%)	1 (3.4%)	0 (0.0%)	
**Time Devoted to Patient Care**				0.187^b^
<50%	10 (18.9%)	3 (10.3%)	7 (29.2%)	
50–74%	25 (47.2%)	14 (48.3%)	11 (45.8%)	
75–100%	18 (33.9%)	12 (41.4%)	6 (25.0%)	

^a^*p*-value from the t-test

^b^*p*-value from Fisher exact Test

^c^*p*-value from Chi-Square Test

### Change in the CCP’s self-efficacy

Following their exposure to the web-based algorithm, CCPs’ self-efficacy for providing smoking prevention and cessation increased significantly (*p* = 0.0029). (Table 3)

### Change in the CCP’s practices

There was also a statistically significant improvement in smoking cessation practices (*p* = 0.0389) among the CCPs following the use of the algorithm. More information is in Table 3.

**Table 3:** Cigarette smoking and smoking cessation self-efficacy and practices among participants (*N* = 53)

	Pre-Algorithm use	Post-Algorithm use	Effect size	*P*-value
**Self-Efficacy** Mean (SD) **Practice** Mean (SD)	27.1 (± 2.44) 26.2 (± 2.08)	28.9 (± 2.10) 27.4 (± 1.46)	0.8 0.7	*0.0029* ^*c*^ *0.0389* ^*c*^

^c^ P-Value from Wilcoxon Signed Rank Test

### The Algorithm’s usability

The average SUS score of the web-based algorithm was 81.5 (± 7.76) in Peru and 84.1 (± 10.26) in Colombia. The overall average SUS score of the algorithm for both countries was 82.9 (± 9.33).

### Qualitative findings

In Colombia, 5 CCPs participated in the first focus group, while 9 CCPs in the second one. In Peru, 8 CCPs participated in each of the two focus groups. Four themes emerged during the qualitative analysis *(1) Limited resources for smoking prevention and cessation in oncology settings, (2) The merits of the algorithm, (3) The challenges with the algorithm, and (4) Suggestions for improving the algorithm tool.*

### Theme 1- limited resources for smoking prevention and cessation in oncology settings

This theme grouped fifteen (15) codes. Among these codes were; deficient prevention services, lack of information on smoking cessation, lack of cessation resources, inadequate time for counseling, poor provider-patient relationship, and lack of smoking cessation culture at their institutions. This theme described the CCPs’ call for the availability of resources that would help them assist their cancer patients with their quitting efforts.“

*“Basically nothing [no availability of smoking prevention and cessation resources]… or well…that we have knowledge of. We only know about the National Addiction Center [in Peru] for any kind of addiction here in Lima”*– Participant 8, 30 years old male Oncology Nurse, Transcript 2, Peru.

*“We do not express to them [cancer patient] the importance of cessation, and we do not follow up with them [cancer patient] throughout this process [of their cessation efforts]”* - Participant 8, 23 years old female Oncology Nurse, Transcript 1, Colombia.

*“[Cancer] patients do not have any information about it [smoking prevention and cessation]” -* Participant 4, 40 years old female Oncology Nurse, Transcript 2, Colombia.”

### Theme 2– Merits of the algorithm

This theme encompassed the value and benefits the CCPs derived from using the algorithm. This theme is made up of thirty-two (32) codes. Some of the codes include; an instructional guide, fosters easy smoking prevention and cessation conversation, fast and easy accessibility, extensive education reach, positive work impact, makes smoking cessation teachable, compatible with technology, simplicity of the tool, comfortable to use, simple language, clarity of tool, step-by-step tool, easy to follow, improved confidence, and time conscious, among others.

*“This is the first time we have known [of] a [web-based algorithm] tool that helps [guide the CCPs to assist their] cancer patients quit smoking” -* Participant 6, 37 years old female Oncology Nurse, Transcript 2, Peru.

*“The language and everything [on the algorithm tool] are simple. There is nothing complicated [with the web-based algorithm].” -* Participant 1, 28 years old female Oncology Nurse, Transcript 2, Colombia.

*“What I liked the most [about the algorithm] is that I had guidelines to follow throughout the smoking cessation counseling. I had the opportunity to see all the steps that are necessary to continue counseling the patient to quit smoking”* - Participant 2, 28 years old female Oncology Nurse, Transcript 2, Columbia.”

### Theme 3– Challenges with the algorithm

In this theme, the CCPs described the challenges they encountered while using the web-based algorithm. There were fifteen (15) codes in this emergent theme. Some codes include; difficulty with QR codes for accessing the algorithm, tool freezing, interruption by incoming phone calls, patient disrespect, poor internet connectivity, and inadequate advice for certain situations.

*“I do not know if it was the type of cell phone [used by the participating CCP], but to me, it [the web-based algorithm] was a little difficult the first time.” -* Participant 4, 40 years old female Oncology Nurse, Transcript 2, Colombia.

*“At the beginning… one loses visual contact with the patient. Because all the time you are [looking at the mobile tool on their mobile phone and not paying attention to the cancer patient]”* - Participant 2, 33 years old female Oncology Nurse, Transcript 2, Peru.”

*My cell phone was not able to scan [the QR code to assess the algorithm], so I had to download an application [QR code scanning app]” -* Participant 5, 54 years old male Oncology Physician, Transcript 2, Colombia.

### Theme 4– Suggestions for improving the algorithm tool

This theme grouped twenty-seven (27) codes and described the modifications the CCPs suggested can be done to improve the subsequent versions of the algorithm. Some of the codes in this theme are; develop an application for immediate accessibility, add pictures/images of the harms of smoking, enable the tool to print reports after counseling, add mechanisms to block phone calls during counseling, include introductory statements, avoid QR codes, include a timer, include statements that motivate palliative care patients, and family inclusion in counseling efforts, among others.

*“I would like to know if it is possible that the tool [algorithm] would be [designed and] free to be downloaded from my cell phone as a mobile application” -* Participant 1, 37 years old female Oncology Nurse, Transcript 1, Peru.

*“It would be ideal to share the materials that were explained to the patient… after the counseling, we can give them a report”* - Participant 3, 33 years old female Oncology Nurse, Transcript 2, Peru.

““*They [cancer patients with low educational levels] will understand [the smoking prevention and cessation message] better through figures [and images]*” - Participant 3, 37 years old female Oncology Nurse, Transcript 2, Columbia.

## Discussion

Our study sample was primarily comprised of oncology nurses, who represent untapped human resources for smoking prevention and cessation interventions as they seem to be eager to participate in behavioral change programs and interventions [32]. Oncology nurses are well positioned to engage cancer patients with smoking prevention and cessation as their interactions with patients can be tapped to institute these interventions [33, 34]. Because cancer care and visits are more comprehensive, hectic, and time-consuming, integrating smoking interventions in these visits may be complex and challenging. However, web-based algorithms like the one tested in our study can help harmonize these processes, thus shortening the time invested in smoking prevention and cessation counseling with the optimization of the treatment outcomes.

Over the years, various smoking prevention and cessation decision guides and algorithms have been developed and used by HCPs and CCPs in the LATAM region [24–26]. However, these guides and algorithms are in static paper-and-pen form, with none digitalized for the HCPs’ use. Therefore, to harmonize and simplify the integration of smoking prevention and cessation services into oncology care, we developed and tested the first smoking prevention and cessation web-based algorithm for CCPs in the LATAM region.

Besides cancer diagnosis and treatment, integrating preventive measures, such as smoking prevention and cessation counseling, is crucial to cancer care [6]. Early identification of smoking behavior in cancer patients and prompt delivery of smoking prevention and cessation are essential for successful cancer treatment outcomes and survivorship [6]. Quitting smoking can help cancer patients live longer, feel healthier overall, and have a lower chance of dying from the disease [6]. Additionally, it can lessen the chance of problems related to treatment and increase the effectiveness of cancer treatment [6].

In the LATAM region, reports from the few smoking prevention and cessation training programs have demonstrated improved self-efficacy and practices among HCPs [8, 10, 11]. For example, the report from the STOP program among CCPs in Peru and Colombia showed these providers’ smoking prevention and cessation self-efficacy and practices to have increased significantly following the hybrid smoking prevention and cessation training [8]. However, in order to sustain the integration of smoking prevention and cessation services over a long term in oncology settings where there is little time to engage in activities other than cancer diagnosis and treatment, it is imperative to keep improving the efficacy and practices of these CCPs. Therefore, the principal aim of this web-based algorithm is to sustain the integration of smoking prevention and cessation in cancer care by continuing to enhance the efficacy and improve the smoking prevention and cessation practices of the CCPs. Following the exposure of the CCPs to our web-based algorithm, the CCPs showed preliminary improvement in their smoking prevention and cessation self-efficacy and practice.

The overall average SUS score for the web-based algorithm in both countries was 82.9 (81.5 in Peru and 84.1 in Colombia), exceeding the benchmark score of 68 for high perceived usability. This shows that the web-based algorithm offers the effectiveness and general ease of use that a CCP needs to navigate the web-based algorithm and provide brief smoking prevention and cessation counseling to their cancer patients. The results of our web-based algorithm are very encouraging, even though there are no reports from digitalized CCPs-operated smoking prevention and cessation algorithms to compare the usability scores.

The paradigm of cancer centers, focused solely on cancer diagnosis and treatment, is now evolving to incorporate preventive services such as smoking cessation due to advancements in digital technology, especially mHealth [35]. Our study demonstrated that the use of web-based algorithms is essential for assisting CCPs in the LATAM region with their efforts to prevent and stop smoking. The CCPs who tested this web-based algorithm emphasized many benefits that come with the use of this web-based algorithm, including simplicity, time efficiency, an instructional guide, and fostering smoking cessation with their cancer patients, among others. The CCPs strongly advised adapting the web-based algorithm into a smartphone application.

### Limitations and strengths

There were some limitations in our study. First, our sample population was restricted to only two countries, which can undermine the generalization of our findings to the LATAM region. The sample population was also majorly composed of oncology nurses, which could have caused other CCPs to miss out on this opportunity. However, oncology nurses are untapped human resources for behavioral change interventions such as smoking prevention and cessation. In addition, there was a lack of diversity in the CCP types represented in our study due to the oncology nurses-dominated sample. As this was a pilot study, future efforts with a larger and more diverse sample of CCPs are granted for determining efficacy, effectiveness, and scale-up for broad dissemination. The lack of reliable internet services and old technology in the CCPs’ countries might have also limited the study. For instance, some CCPs’ devices could not scan QR codes, so they had to download third-party software/apps. As a result, their devices were inundated with spam adverts. Since this was a pilot study, the results of the pre-post effects of the algorithm were only preliminary, as feasibility and efficacy trials to test the efficacy of the algorithm are on the way. Finally, because the CCPs’ responses to the self-efficacy and practice domain questions were self-reported, response bias could be possible.

Despite these limitations, our algorithm was the first smoking prevention and cessation web-based algorithm developed and tested among CCPs in Peru, Colombia, and the LATAM regions. Its results showed high usability and a notable improvement in the CCPs’ smoking cessation self-efficacy and practice. In addition, this web-based algorithm was the seed and foundation for the first-ever web-based decision-tree smartphone app (Decision-T app) for smoking prevention and cessation, designed exclusively for HCPs [23]. At the beta-testing stage, this app showed the potential to increase HCPs’ engagement in offering smoking prevention and cessation behavioral and pharmacotherapy recommendations to their patients briefly and accurately [23].

## Conclusion

Digital algorithms are essential in sustaining smoking prevention and cessation practices integrated into oncology practice. Our web-based algorithm showed high usability and was well-received by the CCPs in Colombia and Peru, with preliminary improvement in their smoking cessation self-efficacy and practice. These results provide justification for the continued development and use of digital algorithms for smoking prevention and cessation in LATAM cancer centers.

### Electronic supplementary material

Below is the link to the electronic supplementary material.

Supplementary Material 1

## Data Availability

The datasets used and/or analyzed during the current study are available from the corresponding author on reasonable request.

## Abbreviations

CCPs: Cancer Care Providers
HCPs: Healthcare Providers
INC: National Cancer Institute in Colombia
INEN: National Institute of Neoplastic Diseases in Peru
LATAM: Latin American
LMIC: Low- and middle-income countries
mHealth: Mobile Health
REDCap: Research Electronic Data Capture
STOP: Smoking Cessation Training Program for the Oncology Practice
SUS: System Usability Scale
