# Supplementary material for: Boosting self-efficacy and improving practices for smoking prevention and cessation among South American cancer care providers with a web-based algorithm
Source: Addict Sci Clin Pract. 2024 May 7;19:36. doi: 10.1186/s13722-024-00462-w (PMC11075359; doi:10.1186/s13722-024-00462-w)
Supplement: Supplementary file 1 — Supplementary Material 1 [file 13722_2024_462_MOESM1_ESM.docx]

**Supplementary Information (SI) Appendix**

**Table S1: Smoking Prevention and Cessation Self-efficacy Questions**

| ***On a scale of 1-5, where 1 = Not at all confident, 2 = Slightly confident, 3 = Somewhat confident, 4 = Confident and 5 = Very confident, how confident are you doing the following:*** | **1** | **2** | **3** | **4** | **5** |
| --- | --- | --- | --- | --- | --- |
| How confident are you that you will be able to recognize cancer patients who are smokers in your clinical practice? |  |  |  |  |  |
| How confident are you that you could help your cancer patients who smoke quit smoking? |  |  |  |  |  |
| How confident do you feel talking about smoking prevention and cessation during your clinical practice without feeling judged by your colleagues/peers? |  |  |  |  |  |
| How confident are you that you will be able to recognize cancer patients who use other tobacco products (e-pipes, cigars, e-cigarettes, hookahs, etc.) in your clinical practice |  |  |  |  |  |
| How confident do you feel about initiating nicotine replacement therapy in your cancer patients who are smokers? |  |  |  |  |  |
| Do you have adequate training to help your cancer patients quit smoking? |  |  |  |  |  |
| Do you need more training in assessing tobacco use and implementing smoking cessation interventions in your patients? |  |  |  |  |  |

**Table S2: Smoking Prevention and Cessation Practice Questions**

| ***On a scale of 1-5 where 1 = Never, 2 = Rarely, 3 = Occasionally, 4 = Sometimes and 5 = Always, how often do you;*** | **1** | **2** | **3** | **4** | **5** |
| --- | --- | --- | --- | --- | --- |
| Do you advise cancer patients who smoke or use other tobacco products to stop smoking or using other tobacco products? |  |  |  |  |  |
| Do you ask cancer patients who smoke or use other tobacco products if they want to quit? |  |  |  |  |  |
| Do you either provide smoking prevention and cessation help to your cancer patients who smoke or refer them to specialized smoking services/programs? |  |  |  |  |  |
| Do you ask your cancer patients during each follow-up visit if they smoke cigarettes? |  |  |  |  |  |
| Do you ask your cancer patients during each visit if they use other tobacco products such as e-pipes, hookahs, e-cigarettes, chewed tobacco, etc? |  |  |  |  |  |
| At each visit, do you reinforce to your cancer patients who smoke or use other tobacco products the importance of smoking cessation? |  |  |  |  |  |
